# Prevalence of malaria and associated clinical manifestations and myeloperoxidase amongst populations living in different altitudes of Mezam division, North West Region, Cameroon

**DOI:** 10.1186/s12936-022-04438-6

**Published:** 2023-01-19

**Authors:** Ntonifor Helen Ngum, Ngahbort Belthine Fakeh, Abongwa Edith Lem, Oumar Mahamat

**Affiliations:** 1 Department of Biological Sciences, Faculty of Science, The University of Bamenda, BP 39, Bambili, N. W. Region Cameroon; 2 African Centre of Excellence for Genomics of Infectious Diseases (ACEGID), Redeemer’s University, Ede, Osun State Nigeria

**Keywords:** Altitude, Malaria, Hemoglobin, White blood cells, Platelet, Myeloperoxidase

## Abstract

**Background:**

Malaria is a growing problem in Africa, with prevalence varies from areas to areas due to several factors including the altitude. This study aimed to investigate the malaria distribution and its relationship with level of some blood parameters and plasma myeloperoxidase (MPO) in population of three localities with different altitudes.

**Methods:**

A total of 150 participants were recruited in each locality and facial body temperature of each was measured using a forehead digital thermometer. Blood samples were collected and used diagnose malaria parasite using the rapid test followed by Giemsa stain microscopy and have the full blood count and MPO level using a colorimetric method.

**Results:**

The overall prevalence of *falciparum* malaria was 34.7%, with no difference between the three communities, but Bambili of high altitude had the highest prevalence (70.7%). A majority of the infected persons had mild malaria, with most cases being asymptomatic (temperature < 37.5 ºC). Patients had significant increase of geometric mean malaria parasite density (GMPD) in Bambili (1755 ± 216 parasites/µL) and Bamenda (1060 ± 2515 parasites/µL of blood) than patients in Santa (737 ± 799 parasites/µL). There was a significant risk to have malaria infection in Bambili (OR = 33.367, p = 0.021) than in Santa (OR = 2.309, p = 0.362). Bambili’ participants of 6–10 years showed a high prevalence of malaria (85.7%). GMPD was significantly different between males (p = 0.010) as well as females (p = 0.000). Participants from Santa (11.2 ± 3.2 g/dL) and Bambili (12.6 ± 2.4 g/dL) had a high haemoglobin concentration than those from Bamenda (10.6 ± 2.1 g/dL). There was a significant difference in the WBC counts and platelet counts among infected participants in the study areas. MPO level had an increasing trend among infected participants in Santa (2.378 ± 0.250), Bambili (2.582 ± 0.482) and Bamenda (2.635 ± 0.466).

**Conclusion:**

The results of the present study demonstrated that altitudinal variations significant impact the risk of population to have malaria with high parasitaemia and may contribute to the malaria prevalence and severity by affecting the haemoglobin concentration, WBC and platelet level and plasma MPO in population.

**Supplementary Information:**

The online version contains supplementary material available at 10.1186/s12936-022-04438-6.

## Background

*Plasmodium falciparum* is the most virulent and deadliest parasite of the human *Plasmodium* species that causes falciparum malaria [1]. This disease is highly prevalent in tropical regions especially sub-Saharan Africa (SSA) and the prevalence in Cameroon has been reported to be about 30% [2]. The occurrence and transmission of malaria parasite in the tropics is influenced by climatic conditions such as temperature and humidity. Changes in altitudinal gradient and attitude towards the use of insecticide-reated bed nets (ITNs) are some underlying factors contributing to the heterogeneity of malaria distribution in endemic communities. The use of ITNs is a World Health Organization (WHO) policy for malaria morbidity control [1, 2] and human attitude towards the use of ITNs affects the morbidity and mortalities associated with the disease. Some studies have reported high malaria transmission in lower altitudinal regions in Cameroon [3], while other studies were of the opinion that altitudinal changes influenced the distribution and density of malaria parasitaemia [4–6]. However, these data are not enough to reach a general conclusion about the altitudinal implication of malaria transmission and distribution in Cameroon due to lack of data points in other endemic areas of the country with apparent variation in altitude and other related climatic factors like temperature and humidity. The availability of data from poorly explored areas in the country is important in reaching a general conclusion about the dynamics of malaria transmission and distribution based on altitudinal differences.

Haemoglobin levels, white blood cell, and platelet counts are frequently affected by malaria infection, they correlate with the pathological effects of malaria parasite in humans [7]. Fluctuating levels of these haematological parameters have been suspected to vary among individuals and people in different altitudinal gradient living with malaria parasite. Data related to this view is limited to the South Western Regions of Cameroon mostly along the slope of Mount Cameroon [5, 8–10].

In addition, immunity against malaria parasite involves a cascade of immune responses including cellular immunity. One important molecule implicated in cellular innate response against invading pathogens is myeloperoxidase (MPO). This is a leucocyte derived enzyme released mostly by neutrophils and monocytes [11] that has been implicated in the formation of leucocyte derived-reactive oxygen species (ROS) reported to be potent against malaria parasite [12]. The direct role of MPO in malaria is still unknown [13], but it is suspected that MPO plays an indirect role in the clearance of malaria parasite through activation of neutrophils and promoting the recruitment of neutrophils and macrophages leading to enhanced proinflammatory response [14, 15] against the parasites.

Information on the distribution of malaria parasites in highlands, semi-highlands, and lowland areas of Mezam division is unknown. Therefore, this study aimed to determine the prevalence of malaria infection using a comprehensive laboratory approach and to identify associated clinical symptoms among a population of different altitudes in the division.

## Methods

### Ethics statement

An administrative authorization for the study was obtained from the Regional Delegate of Public Health for the North West Region. Ethical clearance was obtained from the Institutional Review Board of the Faculty of Health Sciences, The University of Bamenda (N0:2020/0224H/UBa/IRB). The purpose of the study was fully explained to the participants before they took part in the study. They were expected to give their signed informed consent void of coercion. Patients’ results were kept confidential and positive cases referred to meet a medical doctor for treatment (Additional file 1).

### Study area

**Fig. 1:**
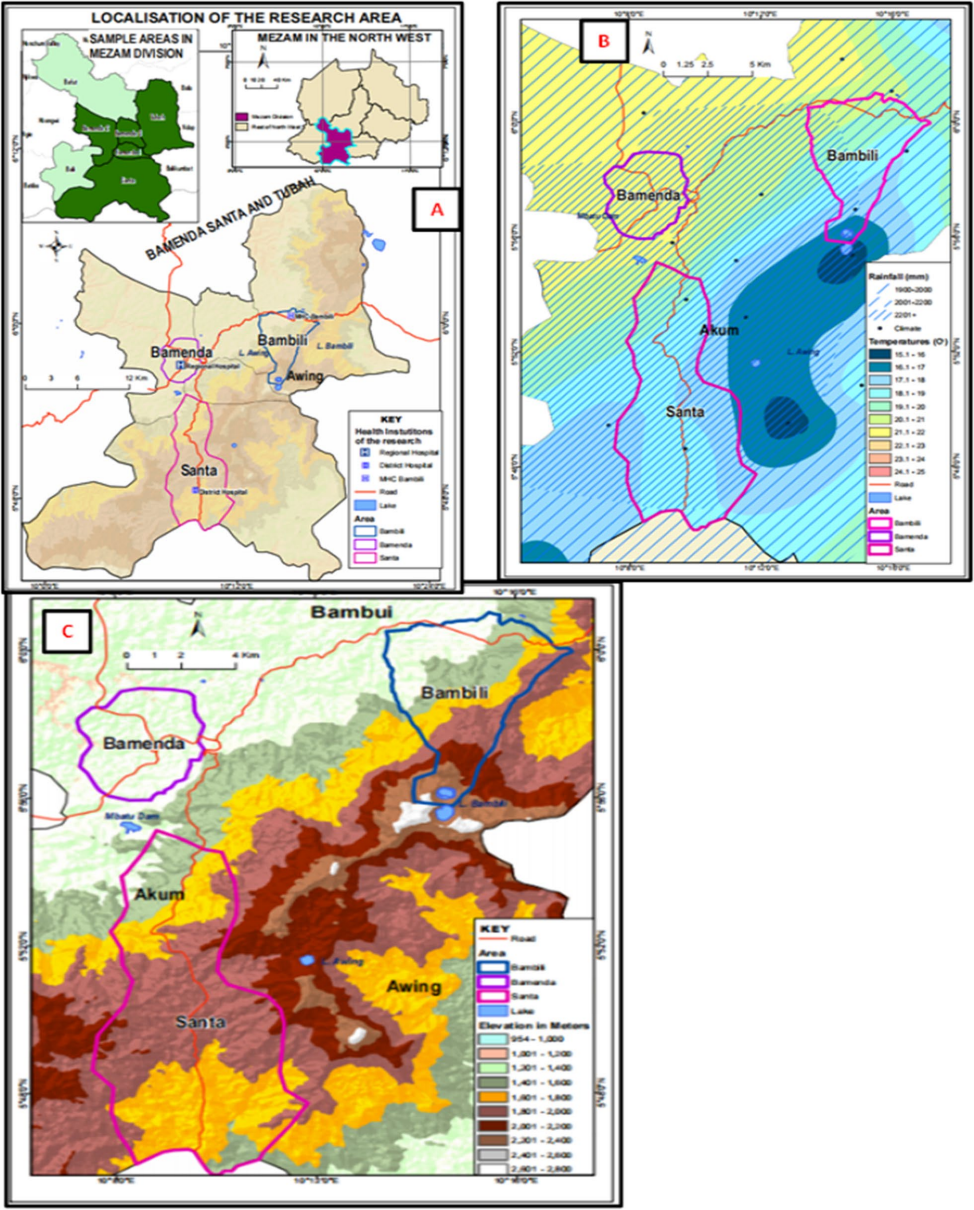
Map of Bambili, Bamenda and Santa in the Mezam Division (February, 2021). **A** Location maps. **B** Map showing climatic conditions of the study area. **C** Map showing altitudinal differences between the study areas

The study was carried out in Bamenda, Bambili, and Santa localities all found in Mezam division of the North West Region of Cameroon. The North West Region lies along the Western highland area of Cameroon experiencing variations in altitude and climatic conditions, and Mezam just like other divisions in Cameroon is endemic for malaria. Cartographic mapping of Mezam (Arte cartogis Nkwen-Bamenda) presents these three localities with varied climatic and geographic settings. Bamenda has higher environmental temperatures (21.1–22 °C) compared to Bambili (16.1–20.0 °C) and Santa (17.1–20.0 °C). The 3 localities experience the same annual rainfall of about 2001 to 2201 mm per year but the Bambili is located at a higher altitudinal gradient (1600–2, 400 m) compared to Bamenda (1201–1400 m) and Santa (1801–2000 m) [16]. This cosmopolitan city having a population of above 39,000 inhabitants is found on a latitude of 10^0^10′0''E and longitude 5^0^56′0''N. Bambili is a village in the Tubah Sub Division and a student residential area located on latitude 10^0^13′0''E and longitude 6^0^0′0''N, while Santa characterized by rural and semi urban settings is located on latitude 10^0^12′0''E and longitude 5^0^48′0''N (Fig. 1). The main economic activity in the rural areas of Mezam is farming while the semi urban and urban areas are commercial centres.

### Study design and period

The study was a hospital based cross-sectional survey conducted in three major health facilities (the Bamenda Regional Hospital, Santa District hospital and the Bambili Community Health Centre) between November 2020 and March 2021. The study involved both laboratory and questionnaire evaluation. The questionnaires were used to collect medical history and demographic information of the participants (Additional file 2). Venous blood was collected from consented participants for parasitological, immunological and haematological analysis. This study involved outpatients of both sexes that were three months and older attending malaria consultation in the above health facilities.

### Sample size and sampling technique

The population sample size was estimated using current proportion of malaria prevalence in Cameroon of 30.3% [17] at 95% confident interval (Z-score = 1.96) using the Cochran formula [18]. The calculated population sample size (323 participants) was adjusted by 40% to 450 participants. The adjusted population was further divided by three based on the three study areas and equal number of participants (150 patients) recruited from each community.

$$ \begin{gathered} {\text{Sample}}\,size\, = \,z^{2} pq/e.^{2} \hfill \\ {\text{Malaria prevalence }}\left( {\text{P}} \right)\, = \,0.{3},{\text{ q}}\, = \,{1}{-\!\!-}{\text{p}}\left( {0.{7}} \right),{\text{ z}}{-\!\!-}{\text{score}}\, = \,{1}.{96},{\text{ error margin}}\left( {\text{e}} \right)\, = \,0.0{5}. \hfill \\ {\text{Sample}}\,size\, = \,\left( {1.96} \right)^{2} \left( {0.3} \right)\left( {0.7} \right)/\left( {0.05} \right).^{2} \hfill \\ {\text{Total sample size }} \approx { 45}0{\text{ participants}}. \hfill \\ \end{gathered} $$

Participants were natives and visitors that had resided in the selected communities for at least three months. All participants gave their written and signed informed consents free of incentives before taking part in the study. Parents and guardians signed the informed consent for minors authorising them to take part in the study. Immunocompromised patients (HIV patience), pregnant women and patients with haemoglobinopathies (sickle cell patients, haemophiliacs, sickle cell carriers), and those on malaria treatment or had taken malaria treatment in the last one month were disqualified from the study.

### Data collection

A well-structured questionnaire written in English language was used to screen and collect demographic information (sex, age, and residency) about the participants. At enrolment, patients facial skin temperatures were measured using an infrared forehead digital thermometer (HTD8813C, Hetaida Technology Co. Ltd, China) and the results recorded in degree Celsius. Pyrexia related to malaria was defined as temperatures > 37.5^0^C [19].

### Laboratory sample collection and malaria diagnosis

From each recruited patient, at least 2 mL of venous blood was collected into Ethylenediaminetetraacetic Acid (EDTA) tubes using a vacutainer and transported in a safe flask to the parasitology laboratory of the health facility in the various study areas. Recruited participants were first screened for malaria parasite using a rapid diagnostic test kit (Care Start MR19B69, China) conjugated with *P. falciparum* histidine protein II (PfHRP II) for specific identification of *P. falciparum*.

For microscopic examination, both thin and thick blood films were prepared on labelled grease free glass slides using 1µL of blood from each EDTA test tube. The thin films were fixed using methanol for 10 s. Later, both films were stained using 10% Giemsa for 10 min, then carefully washed and the slides air dried using a hair dryer (Xtava, Allure Co. Ltd, USA). The oil immersion (× 100) objective of a light microscope was used to observe the slides. The bench aid for the diagnosis of malaria parasite was used for the identification and confirmation of any malaria parasite [20]. Malaria parasitic species were appreciated and confirmed on the thin films while the thick films were used for the determination of malaria parasite density per microlitre (µL) of blood. Blood stages (trophoziotes) of the parasite were counted against 200 white blood cells (WBCs) assuming total WBC count of 8, 000 cells/µL of blood [21]. Up to 100 high power fields were observed before declaring a slide negative. The malaria parasite (MP) density was further classified as mild (MP < 1000/µL of blood), moderate (MP between 1000 and 4999/µL of blood) and severe (MP ≥ 5000/µL of blood) [5]. The density of the parasite was calculated as follows:

*Parasite density* = *[counted parasites/total WBC counted (200)] x [*8000WBCs].

### Determination of haemoglobin concentration, total white blood cells and platelet counts

Haemoglobin concentration, total WBCs and platelet counts were obtained after performing complete blood count using a haematological analyser machine (URIT-3300, Urit medical Electronic Company Ltd China), following the protocol of the manufacturer. Blood cell indices such as haemoglobin concentration, total platelets count, WBC count were recorded. Haemoglobin concentration < 11 g/dL of blood was used to define anaemia in the patient. Anaemia was further classified as mild (Hb 10.0–10.9 g/dL), moderate (Hb 7–9.9 g/dL) and severe (Hb < 7 g/dL) [21]. WBC count of > 10,000 cells/μL was classified as Leucocytosis while Leucopenia was WBC count < 4000 cells/μL.

### Assessment of plasma myeloperoxidase level

Myeloperoxidase was assessed using a colometric method as adopted by Oumar et al. [22] with a few modifications. Briefly, one hundred microlitre of phenylenediamine solution and 0.002% hydrogen peroxide in phosphate-citrate buffer (P^H^ = 5.0) were mixed with equal volumes (100 µL) of serum on a microplate. The reaction was stopped after 10 min using 0.1 M sulphuric acid and absorbance was recorded at 490 nm using an ELISA reader (ELISA analyzer, Urit-660, China).

### Statistical analysis

The cleaned data was uploaded into the statistical package for social sciences (SPSS) version 23 (IBM SPSS Inc. Chicago, IL, USA) for statistical analysis (Additional file 3). The Pearson Chi-Square test was used to explore proportions between groups. One Way ANOVA (Analysis of Variance) and the independent student t-test or the Turkey HSD was used as a post hoc test for multiple comparisons of means between variables such number of blood cells, haemoglobin level and myeloperoxidase level. The Mann–Whitney U test and Kruskal–Wallis Test were used to analyse the mean differences between groups for non-parametric data such as prevalence. The binary logistic regression with non-adjusted odd ratios was used to evaluate some predictors of malaria in the study. The malaria parasite densities were log transformed in base 10 before applying the ANOVA and Turkey HSD multi-comparison tests to explore the difference in the means of the malaria parasite densities between the geographic areas. The cut off point for statistical significance between groups was set at probability level *p* ≤ 0.05.

## Results

### Baseline characteristics of the study population

A total of 450 patients were recruited into the study and females recorded the highest participatory rate in all the study areas [60.9% (274/450)]. Most participants [27.6% ($$124/450)]$$ were between the ages of 21–35 years (Table 1) and at enrolment, majority of patients in all the study areas were afebrile [88.2% ($$397/450)]$$ with just 11.8% (53/450) of the total population presenting with pyrexia and patients in Bamenda recording the highest pyrexia rate [16.7% ($$25/150)]$$. The overall mean temperature was 37.0 ± 0.5^0^C. About 19.3% ($$87/450)$$ of the entire population was anaemic and patients in Bamenda recorded more cases of anaemia [28.7% ($$43/150)]$$.

**Table 1:** Baseline characteristics of the study population based on demographic, clinical and climatic parameters

Parameters	Location			Total
	Bamenda n (%)	Santa n (%)	Bambili n (%)	
Total	150 (33.3)	150 (33.3)	150 (33.3)	450 (100)
Sex				
Males	65 (43.3)	54 (36.0)	57 (38.0)	176 (39.1)
Females	85(56.7)	96 (64.0)	93 (62.0)	274 (60.9)
Mean (± SD) age	30.2 ± 24.7	31.2 ± 23.3	24.7 ± 19.7	28.7 ± 22.8
Age grp (years)				
$\le$ 5	43 (28.7)	31 (20.7)	31 (20.7)	105 (23.3)
6–10	9 (6.0)	1 (0.7)	7 (4.7)	17 (3.8)
11–20	4 (2.7)	21(14.0)	28 (18.7)	53 (11.8)
21–35	29 (19.3)	42 (28.0)	53 (35.3)	124 (27.6)
36–60	45 (30.0)	36 (24.0)	20 (13.3)	101 (22.4)
≥ 61	20 (13.3)	19 (12.7)	11(7.3)	50 (11.1)
Mean (± SD) temp. (°C)	37.1 ± 0.4	36.8 ± 0.4	37.0 ± 0.4	37.0 ± 0.5
Fever status				
Febrile	25 (16.7)	10 ( 6.7)	18 (12.0)	53 (11.8)
Afebrile	125 (83.3)	140 (93.3)	132 (88.0)	397 (88.2)
Anaemia prevalence (Hb < 11 g/dL)	43 (28.7)	21 (14.0)	23 (15.3)	87 (19.3)

### Spatial distribution of malaria infection

Out of the 450 participants in the study, 156 tested positive for malaria giving a prevalence of 34.7% (Table 2). The distribution *of Plasmodium* spp between the three geographical areas was significantly different (χ^2^ = 103.26; p = 0.016). Patients from Bambili registered more positive cases of malaria [70.7% ($$106/150)]$$ while Santa had the least [6.7% $$(10/150)]$$. Out of the 43,7% infected patients in the three localities, 87.2% ($$136/156)$$ had asymptomatic malaria though the differences were not statistically significant (p = 0.169) when compared to asymptomatic cases in the three localities. Within the localities, only in patients from Santa, the prevalence of asymptomatic malaria was significantly higher than symptomatic malaria (χ^2^ = 10.000, p = *0.019*). The results (Table 2) showed that there was no significant difference in malaria severity level between the different localities, most patients in Bamenda [65.0% (26/40)] and Santa [80.0% (8/10)] were mildly infected with malaria, while in Bambili, the prevalence of moderate malaria [64.4% [$$68/106)]$$ was the highest. Patients in Bambili and Bamenda significantly (F = 6.065; *p* = *0.003*) had higher geometric mean parasite density (GMPD ± SEM) of 1755 ± 216 µL and 1060 ± 2515 µL of blood) respectively compared to patients in Santa (Table 2).

**Table 2:** Prevalence, malaria category and parasite density following the different geographic settings

Variables	Study area			Total	Significant difference
	Bamenda	Santa	Bambili		
Number examined	150	150	150	450	χ^2^ = 29.286; p = 0. 398
Prevalence n (%)					
Symptomatic (T ≥ 37.6^0^C)	5 (12.5)	1 (10.0)	14 (13.2)	20 (12.8)	
Asymptomatic (T ≤ 37.5^0^C)	35 (87.5)	9 (90.0)	92 (86.8)	136(87.2)	χ^2^ = 89.88 p = 0. 169
Total	40 (26.7)	10 (6.7)	106 (70.7)	156(34.7)	χ^2^ = 103.26; p = 0. 016
Chi-Square	χ^2^ = 12.571 p = 0.322	χ^2^ = 10.000 p = *0. 019*	χ^2^ = 54.773 p = 0. 074	χ^2^ = 54.773 p = 0. 074	
Malaria severity n (%)					
Mild	26 (65.0)	8 (80.0)	26 (24.5)	60 (38.5)	χ^2^ = 21.44; p = 0. 091
Moderate	9 (22.5)	1 (10.0)	68 (64.4)	78 (50.0)	χ^2^ = 52.41; p = 0.533
Severe	5 (12.5)	1 (10.0)	12 (11.3)	18 (11.5)	χ^2^ = 17.20; p = 0. 509
GMPD ± SEM/µL (Range)	1060 ± 2515^a^ (400–92,000)	737 ± 799^b^ (400–8000)	1755 ± 216^c^ (320–13,640)	1459 ± 664 (320–92,000)	F = 6.065; *p* = *0.003*
Post Hoc test (Tukey HSD)	^ab^*p* = 0.56	^ac^*p* = 0, 02	^bc^*p* = 0.02		

The supscripts letters on the p value (ANOVA or Tukey-HSD) indicate the significance for the parameters value with the same letters

*GMPD *Geometric mean parasite density, *SEM *standard error of mean, *T *temperature

### Relationship between altitude and the prevalence of malaria in the different geographical settings

Table 3 presents the risks to have malaria in Santa and Bambili with reference to the infection in Bamenda. There was a significant risk to have malaria infection in Bambili (OR = 33.367, 95%C. I: 1.691–658.411, p = 0.021) while the risk to have malaria in Santa was not statistically significant (OR = 2.309, 95%C. I: 0.382–13.958, p = 0.362).

**Table 3:** Logistic binary regression

Areas (Altitude in m)	Number examined	Dependent variable: *P. falciparum* malaria			
		S.E	Wald	Sig	Odd ratio (95% CI)
Bamenda (1201–1400)	150		5.468	*0.054*	Reference
Santa (1801–2000)	150	0.918	0.830	0.362	2.309 (0.382–13.95)
Bambili (1600–2400)	150	1.522	5.314	*0.021*	33.367 (1.691–658.41)

### Influence of sex and age on the prevalence and density of malaria parasite in the different geographical settings

Table 4 shows the age and gender base prevalence of Malaria in the study areas. There were no significant difference in the distribution of malaria between males and females in each geographical setting, although males recorded a higher prevalence of malaria in the entire study [38.6% (68/176)] than females [32.1% (88/274)]. Heightened malaria prevalence was observed among males in Bamenda [33.8% (22/65)] and Santa [11.1% (6/54)], while in Bambili the prevalence was higher in females [71.0% (66/93)]. There was a sound significant difference (Mann Whitney U test, *p* = *0.007*) in the GMPD ± SEM between males and females in the entire study. The density of malaria parasite was higher in females GMPD ± SEM (1691 ± 265 µL) than males (1205 ± 1487 µL). Following the different areas, a significant (Mann Whitney U test, *p* = *0.009*) difference was observed only in Bambili, with high prevalence in females (2088 ± 305 µL) than males (1317 ± 237 µL). There was a high significant difference in the mean malaria parasitaemia in each sex group across the three study areas with male patients in Bamenda and Bambili recording higher values (Kruskal Wallis test*, p* = *0.010*) than male patients in Santa. Conversely female patients in Santa and Bambili recorded significantly (Kruskal Wallis test*, p* = *0.000*) higher parasitaemia than female patients in Bamenda (Table 3).

**Table 4:** Prevalence and density of malaria parasite in relation to sex and age in the different geographical settings

Variables	Malaria prevalence % (n)				Significant level	GMPD ± SEM				Significant difference
	Bamenda 150	Santa 150	Bambili 150	Total 450		Bamenda	Santa	Bambili	Total	
Sex										
Males	33.8 (22)	11.1(6)	70.2 (40)	38.6 (68)	χ^2^ = 58.179 *p* = 0.796	1332 ± 4498 (400–92,000)	458 ± 43 (400–600)	1317 ± 237 (320–5600)	1205 ± 1487 (320–92,000)	*p* = *0.010*
Females	21.2(18)	4.2 (4)	71.0(66)	32.1(88)	χ^2^ = 57.433 *p* = 0.422	802 ± 441 (400–8000)	1505 ± 1812 (400–8000)	2088 ± 305 (320–13,640)	1691 ± 265 (320–13,640)	*p* = *0.000*
Sig. level	χ^2^ = 12.98 *p* = 0.295	χ^2^ = 4.444 *p* = 0. 217	χ^2^ = 36.272 *p* = 0. 681	χ^2^ = 46.465 *p* = 0. 453		*p* = 0.421	*p* = 0.476	*p* = *0.009*	*p* = *0.007*	
Age group (yrs)										
0–5	32.6(14)	9.7(3)	74.1(23)	38.1(40)	χ^2^ = 34.509 *p* = 0. 715	654 ± 136 (400–2000)	400 ± 0.00 (400–400)	2635 ± 666 (760–13,640)	1405 ± 448 (400–13,640)	*p* = *0.000*
6–10	44.4(4)	0.0(0)	85.7(6)	58.8(10)	χ^2^ = 7.917 *p* = 0. 340	1911 ± 6272 (400–26,000)	0	860 ± 203 (400–1800)	1184 ± 2509 (400–26,000)	*p* = 0.748
11–20	25.0(1)	14.3(3)	75.0(21)	47.2(25)	χ^2^ = 13.265 *p* = 0.962	800 ± 0.00 (800–800)	987 ± 1169 (400–4000)	1142 ± 238 (320–4120)	1106 – 233 (320–4120)	*p* = 0.732
21–35	17.2(5)	4.8(2)	62.3(33)	32.3(40)	χ^2^ = 35.065 *p* = 0. 767	1067 ± 1059 (400–6000)	1789 ± 3800 (400–8000)	1793 ± 349 (320–8400)	1680 ± 320 (320–8000)	*p* = 0.493
36–60	24.4(11)	5.6(2)	70.0(14)	26.7(27)	χ^2^ = 38.513 *p* = *0. 054*	1325 ± 8243 (400–92,000)	490 ± 100 (400–600)	2245 ± 551 (1000–8000)	1618 ± 3353 (400–92,000)	*p* = *0.030*
≥ 61	25.0(5)	0.0(0)	81.8(9)	28.0(14)	χ^2^ = 8.919 *p* = 0.349	1652 ± 7823 (400–4000)	0	1715 – 406 (400 – 3600)	1692 ± 2749 (400–40,000)	*p* = 0.347
Sig. level	χ^2^ = 51.09 *p* = 0. 627	χ^2^ = 9.722 *p* = 0. 373	χ^2^ = 238.715 *p* = 0. *053*	χ^2^ = 267.677 *p* = *0. 027*		*p* = 0.807	*p* = 0.444	*p* = *0.010*	*p* = 0.575	

The Mann Whitney U and Kruskal Wallis tests were used to explore means between two and more than variables respectively

*n* number of cases, *GMPD ± SEM* Geometric mean parasite density ± standard error of mean

There was a significant distribution of *P. falciparum* between age groups in the overall study (χ^2^ = 267.677, *p* = *0.027*). In the different study areas, Bambili had a significant difference in prevalence between the age groups (χ^2^ = 238.715*, p* = *0.053*), with patients 6–10 years old showing higher prevalence. For each age group across the three study areas, only patients aged 36–60 years old recorded significant distribution of malaria parasite (χ^2^ = 38.513*, p* = *0.054*) with patients in Bambili having 70.0% (14/20), Bamenda 24.4% (11/45), and Santa 5.6% (2/36) Table 4.

### Influence of malaria infection on the haematological symptoms in the population

As shown on Table 5, the haemoglobin concentration levels of patients residing in Bamenda were lower (11.3 ± 3.4 g/dL) compared to those of Santa and Bambili who recorded significant (F = 1.875, *p* = *0.145*^*svy*^) higher mean (± SD) haemoglobin concentrations of 11.9 ± 1.5 g/dL and 11.8 ± 3.5 g/dL of blood respectively, even though the post hoc test did not show any significant difference (p = *0.145*^*svy*^). In patients with malaria, a significant difference was observed in Hb in the three study areas (p = 0.004). Patients that tested positive for *P. falciparum* in Bamenda had lower mean haemoglobin concentrations (Hb 10.6 ± 2.1 g/dL) compared to patients’ resident in Bambili (Hb 12.6 ± 2.4 g/dL). A significant difference was also observed between Hb in the malaria negative patients (p = 0.001^jmp^). Patients that tested negative for *P. falciparum* in Bambili had lower Hb (Hb 9.9 ± 4.7 g/dL) as compared to patients in Bamenda (Hb 11.5 ± 3.7 g/dL, p = 0.843) and Santa (Hb 11.9 ± 1.3 g/dL, p = 0.000). There was a significant difference in the haemoglobin concentration levels between patients that were positive for *P. falciparum* as compared to those that tested negative in Santa (t = 1.544, *p* = *0.000*) and Bambili (t = − 4.601, *p* = *0.000*).

**Table 5:** Variation of haemoglobin levels, white blood cells and platelets following *P. Falciparum* infection in different geographical settings

Location	Blood parameters	Malaria status		Total	Sig. level	Normal range
		Positive	Negative			
Bamenda	Hb conc. (g/dL)	10.6 ± 2.1^a^ (n = 40)	11.5 ± 3.7^j^ (n = 110)	11.3 ± 3.4^s^ (N = 150)	t = 1.433, *p* = 0.843	11.0–15.0
	WBCs × 10^3^µL	6.1 ± 3.8^b^	6.1 ± 2.9^k^	6.1 ± 3.2^t^	t = 0.003, *p* = 0.382	4.0–10.0
	Plateletx 10^3^µL	302 ± 154^c^	352 ± 117^l^	338 ± 129^u^	t = 2.114, *p* = *0.031*	100–300
Santa	Hb conc. (g/dL)	11.2 ± 3.2^d^ (n = 10)	11.9 ± 1.3^m^ (n = 140)	11.9 ± 1.5^v^	t = 1.544, *p* = *0.000*	11.0–15.0
	WBCs × 10^3^ µL	9.6 ± 6.9^e^	9.2 ± 3.5^n^	9.2 ± 3.8^w^	t = − 0.544, *p* = *0.042*	4.0–10.0
	Plateletx 10^3^ µL	286 ± 218^f^	251 ± 86^o^	253 ± 99^x^	t = − 1.090, *p* = *0.000*	100–300
Bambili	Hb conc. (g/dL)	12.6 ± 2.4^ g^ (n = 106)	9.9 ± 4.7^p^ (n = 44)	11.8 ± 3.5^y^	t = − 4.601, *p* = *0.000*	11.0–15.0
	WBCs × 10^3^ µL	4.5 ± 2.1^ h^	4.8 ± 1.9^q^	4.6 ± 2.1^z^	t = 0.501, *p* = 0.162	4.0–10.0
	Plateletx 10^3^ µL	465 ± 145^i^	465 ± 98^r^	465 ± 132^σ^	t = − 0.027, *p* = *0.006*	100–300
Significant difference (ANOVA)		*p* = *0.000*^*adg*^* p* = *0.000*^*beh*^* p* = *0.000*^*cfi*^ F = 10.347 F = 13.987 F = 20.508	*p* = *0.001*^*jmp*^* p* = *0.000*^*knq*^* p* = *0.000*^*lor*^ F = 7.366 F = 49.149 F = 85.828	*p* = *0.145*^*svy*^* p* = *0.000*^*twz*^* p* = *0.000*^*ux*σ^ F = 1.875 F = 87.390 F = 117.565		
Post Hoc Test (Tukey HSD)		0.779^ad^; *0.000*^*ag*^; 0.188^dg^ *0.006*^*be*^*; 0.018*^*bh*^*; 0.000*^*eh*^ 0.951^cf^; *0.000*^*ci*^; *0.001*^*fi*^	0.542^jm^; *0.009*^*jp*^; *0.000*^*mp*^ *0.000*^*kn*^*; 0.036*^*kq*^*; 0.000*^*nq*^ *0.000*^*lo*^*; 0.000*^*lr*^*; 0.000*^*or*^	0.179^*sv*^; 0.265^sy^; 0.974^vy^ *0.000*^*tw*^*; 0.000*^*tz*^*; 0.000*^*wz*^ *0.000*^*ux*^*; 0.000*^*u*σ^; 0.000^xσ^		
TOTAL	Hb conc. (g/dL)	12.0 ± 2.5 (n = 156)	11.5 ± 3.1 (n = 294)	11.7 ± 2.9 (N = 450)	t = − 1.806, *p* = 0.189	11.0 – 15.0
	WBCs × 10^3^ µL	5.3 ± 3.4	7.4 ± 3.6	6.7 ± 3.6	t = 6.125, *p* = *0.001*	4.0–10.0
	Platelet × 10^3^ µL	412 ± 170	321 ± 126	352 ± 149	t = − 6.493, *p* = *0.000*	100–300

The supscripts letters on the p value (ANOVA or Tukey-HSD) indicate the significance for the parameters value with the same letters

*SD* standard deviation, *WBC* White blood cells, *Hb* haemoglobin concentration

There was equally a significant (F = 87.390, *p* = *0.000*^*twz*^) difference in the mean white blood cell count among patients resident in the different geographical study areas. Participants in Santa (9.2 ± 3.8 × 10^3^µL, p = 0.000^tw^) and Bamenda (6.1 ± 3.2 × 10^3^µL, p = 0.000^tz^) had higher mean WBC counts than patients in Bambili (4.6 ± 2.1 × 10^3^µL, p = 0.000^wz^). There was a significant difference in the level of WBC in both positive (F = 13.987, *p* = 0.000^beh^) and negative (F = 49.149, *p* = *0.000*^knq^) patients in all the three study areas. In the positive patients, the WBC level was higher in Santa as compared to Bambili (p = 0.000^eh^) and Bamenda (p = 0.006^eb^). It was also observed that patients from Bamenda had higher level of WBC count compared to those from Bambili (p = 0.018^bh^). Only patients in Santa showed a significant difference (t = − 0.544, *p* = *0.042*) in the mean WBC count between patients that tested positive (9.6 ± 6.9 × 10^3^ µL) and negative (9.2 ± 3.5 × 10^3^ µL for *P. falciparum* (Table 5).

In addition, the results showed a significantly (F = 117.565, *p* = *0.000*^*ux*σ^) increasing trend in the mean platelet count among patients in Santa (253 ± 99 × 10^3^µL), Bamenda (338 ± 129^u^ × 10^3^ µL) and Bambili (465 ± 132 × 10^3^ µL). A similar trend was observed among patients that were positive (F = 20.508, *p* = *0.000*^*cfi*^) for *P. falciparum* and among those that were negative (F = 85.828, *p* = *0.000*^*lor*^) between the study areas (Table 5). The post hoc analysis also revealed significant variations in the mean platelet count within the localities among those that had malaria and among those that were not infected (Table 5). There were significant differences in the mean platelet counts between patients that were positive for malaria parasite and those that were negative in Bamenda (t = 2.114, *p* = *0.031*), Santa (t = − 1.090, *p* = *0.000*) and Bambili (t = − 0.027, *p* = *0.006*).

### Influence of age and sex on the occurrence of anaemia in the population

Out of the 450 patients examined, 87 (19.3%) of them were anaemic and there was a significant (χ^2^ = 93.460, *p* = *0.004*) variation in anaemia prevalence based on geographical location. Patients from Bamenda recorded the highest prevalence (28.7% [$$43/150])$$ and the least was among patients in Santa (14.0% [$$21/150])$$. Females of the three study areas showed a significant difference in the prevalence of anaemia (χ^2^ = 58.346, *p* = *0.006*) with females in Bamenda recording the highest (25.9 [$$22/85$$]) prevalence of anaemia compared to Santa and Bambili (Table 6). Males however recorded a significantly (χ^2^ = 45.765, *p* = *0.033*) higher prevalence of anaemia (21.6%) than females (17.9%) in the whole study. Males in Bamenda significantly (χ^2^ = 33.662, *p* = *0.053*) registered a higher prevalence 32.3% ($$21/65$$) of anaemia when compared to females (25.9% [$$22/65$$]). With respect to age, there was a significant difference in anaemia prevalence in the entire study (χ2 = 240.679, p = 0.000).

**Table 6:** Prevalence of anaemia based on age and sex in the study populations

Variables	Bamenda % (R)	Santa % (R)	Bambili % (R)	Total % (R)	Significant level
Sex					
Males	$32.3(21/65)$	$16.7(9/54)$	$14.0(8/57)$	$21.6(38/176)$	χ^2^ = 54.346, *p* = 0.186
Females	25.9 (22/85)	$12.5(12/96$)	$16.1(15/93)$	$17.9(49/274)$	χ^2^ = 58.346, *p* = *0.006*
Total	28.7 (43/150)	$14.0(21/150)$	$15.3(23/150)$	$19.3(87/450)$	χ^2^ = 93.460, *p* = *0.004*
Significant difference	χ^2^ = 33.662, *p* = *0.053*	χ^2^ = 10.792, *p* = 0.461	χ^2^ = 14.183, *p* = 0.077	χ^2^ = 45.765, *p* = *0.033*	
Age group (years)					
0–5	$32.6(14/43)$	$19.4(6/31)$	$16.1(5/31)$	$23.8(25/105)$	χ^2^ = 32.679, *p* = 0.172
6–10	$11.1(1/9)$	0	0	$5.9(1/17 )$	–
11–20	$25.0(1/4)$	$14.3(3/21)$	$10.7(3/28)$	$13.2(7/53)$	χ^2^ = 14.000, *p* = 0.301
21–35	$20.7(6/29)$	$14.3(6/42)$	$11.3(6/53)$	$14.5(18/124$)	χ^2^ = 32.679, *p* = 0.289
36–60	$35.6(16/45)$	$5.6(2/36)$	$25.0(5/20$)	$22.8(23/101$)	χ^2^ = 21.419, *p* = 0.495
≥ 61	$25.0( 5/20$)	$21.1(4/19)$	$36.4(4/11$)	$26.0(13/50$)	χ^2^ = 26.000, *p* = 0.100
Significant difference	χ^2^ = 164.735, *p* = *0.001*	χ^2^ = 47.250, *p* = 0.341	χ^2^ = 32.008, *p* = 0.366	χ^2^ = 240.679, *p* = *0.000*	

*R (Ratio)* Observed cases over the number examined/Total number of patients examined)

Patients that were ≤ 5 years old and those of 61 years and above registered significantly high anaemia prevalence of (23.8% [$25/105$]) and (25.9% [$22/65$]) respectively compared to other age groups in the entire study. A significant difference between age groups was also observed only in Bamenda where patients aged 0–5 years (32.6% [$14/43$]) and 36–60 years (35.6% [$16/45$]) were significantly (χ^2^ = 164.735, *p* = *0.001*) more anaemic.

### Influence of malaria status and severity on the level of plasma myeloperoxidase in the different geographical settings

As shown on Table 7, irrespective of the study area, patients who tested positive for *P. falciparum* had significantly (t = − 2.803, *p* = *0.003*) higher mean myeloperoxidase levels (2.554 ± 0.437) than patients who were negative (2.117 ± 0.862). With respect to study areas, malaria positive and negative patients showed a significant difference in MPO (p = 0.003). Participants in Santa who tested positive for *P. falciparum* had highly significant (t = − 2.056, *p* = *0.000*) MPO levels (2.378 ± 0.250) than participants that tested negative (1.721 ± 0.980).

**Table 7:** Plasma myeloperoxidase levels in the different populations based on the malaria status and severity in the different geographical settings

Variables	Mean ± SD serum myeloperoxidase level			Total	ANOVA (One way)	Tukey-HSD Post Hoc test Significance
	Bamenda	Santa	Bambili			
Malaria parasite status						
Positive	2.635 ± 0.466^a^	2.378 ± 0.250^b^	2.582 ± 0.482^c^	2.554 ± 0.437	F = 1.112, *p* = 0.338^abc^	0.327^ab^; 0.939^ac^; 0.461^bc^
Negative	2.553 ± 0.364^d^	1.721 ± 0.980^e^	2.726 ± 0.185^f^	2.117 ± 0.862	F = 3.734, *p* = *0.041*^*def*^	*0.055*^*de*^; 0.956^df^; 0.227^ef^
Total	2.604 ± 0.426^ g^	2.007 ± 0.812^ h^	2.595 ± 0.461^i^	2.402 ± 0.648	F = 7.703, *p* = *0.001*^*ghi*^	*0.002*^*gh*^; 0.999^gi^; *0.004*^*hi*^
Independent t—test	t = − 0.437 *p* = 0.498	t = − 2.056 *p* = *0.000*	t = 0.410 *p* = 0.393	t = − 2.803 *p* = *0.003*		
Malaria severity level						
Mild (MP < 1000µL)	2.611 ± 0.409^j^	2.420 ± 0.252^ k^	2.485 ± 0.533^ l^	2.515 ± 0.399	F = 0.515, *p* = 0.605	0.591^jk^; 0.807^jl^; 0.949^kl^
Moderate (MP:1000–4999)	2.669 ± 0.659^ m^	2.056 ± 0.120^n^	2.670 ± 0.472^o^	2.634 ± 0.519	F = 0.626, *p* = 0.549	0.615^ mn^; 0.486^mo^, 0.845^no^
Severe (MP ≥ 5000 µL)	2.653 ± 0.201^p^	2.359 ± 0.024^q^	2.285 ± 0.340^r^	2.432 ± 0.195	F = 0.378, *p* = 0.634	0.862^pq^, 0.861^pr^, 0.672^qr^
ANOVA (One way)	F = 0.361, *p* = 0.241	F = 0.756, *p* = 0.647	F = 0.649, *p* = 0.482	F = 7.703 *p* = 0.001		

The supscripts letters on the p value (ANOVA or Tukey-HSD) indicate the significance for the parameters value with the same letters

*ANOVA* Analysis of variance between variables, *SD* standard deviation

Comparing the levels of MPO between the study areas, there was a significant variation (F = 7.703, *p* = *0.001*^*ghi*^) in the MPO levels. The MPO level was significantly lower in Santa (2.007 ± 0.812), as compared to Bamenda (2.604 ± 0.426^ g^) and Bambili (2.595 ± 0.461^i^). In malaria positive patients, there was no significant difference (F = 1.112, *p* = 0.338^abc^) in MPO when comparing the three different study areas. In patients who tested negative for *P. falciparum,* there was a significant variation (F = 3.734, *p* = *0.041*^*def*^) in the mean MPO levels among patients in the different geographic area, with a low MPO in participants of Santa than those of Bamenda and Bambili, but the difference was significant only in Bamenda (p = *0.05*^*de*^). Independent of the geographic areas, there was a significant difference between patients with mild, moderate and severe malaria (F = 7.703, *p* = *0.001*), but no difference was observed with each area with respect to the malaria categories as well as when comparing participants of the different study areas within the same malaria severity level.

## Discussion

The study aimed at assessing the influence of altitude on malaria prevalence, parasitaemia and comorbidity. The study was carried out in three different localities: Santa, Bambili and Bamenda with altitudes of 1801–2000 m, 1600–2400 m and 1201–1400 m above sea level, respectively. The overall prevalence of malaria among participants in the entire study was 34.7%. A similar community based study conducted along the slope of mount Cameroon in the South West Region showed lower (33.8%) results [5]. These results were also higher than the national prevalence (30.3%) of malaria in Cameroon [2]. The overall prevalence of malaria was significant different among participants in Santa, Bamenda, and Bambili with 6.7%, 26.7%, and 70.7% respectively. This variation in malaria prevalence may be due to the differences in the target population notably their knowledge of the disease, but not due to the apparent difference in altitude of the three localities. This was in contrast to results of similar studies conducted in Tanzania [4] and in the South West Region of Cameroon [5] that reported high prevalence and transmission of malaria in lowland areas compared to high land areas. The three localities in which this study was conducted are found in the Western Highland area of Cameroon. Though these localities show some variations in climatic conditions (altitude, environmental temperatures), they however experience common intersperse topographic features with overlapping climatic conditions that apparently show no clear cut stiff altitudinal gradient between them. This may be insignificant to influence a bias in the distribution and abundance of the mosquito vector for malaria parasite between these communities hence possibly explain the absence of direct correlation between the malaria prevalence among the populations and the altitude of these localities. However, patients in Bambili of a higher altitude than Bamenda, recorded a higher prevalence of malaria than patients in Santa and this had a similar outcome with the results of [23] in the South West Region of Cameroon that reported higher prevalence of malaria among populations of higher altitudes. This might suggest that changes in environmental conditions and human activities that favour the adaptation and development of the mosquito vector in higher land areas, Bambili is also a student residential area that is currently experiencing a population boom. Also, Bamenda city that is a transit to this community is marked with social activities in the region coupled with the influx of inhabitants from the different villages in the North West Region due to the Socio-political crises. The attitude of the inhabitants such as poor dressing exposing body parts to the bite of mosquitoes, and keeping late evening hours drinking that coincide with the biting period of mosquitoes maybe contributing factors to the high prevalence of malaria in these communities irrespective of their apparent altitudinal differences.

The malaria parasite density in population in the different areas was significantly different in males as well as in females. In males, the density of malaria parasite was significantly lower in Santa compared to that in Bamenda and Bambili which did not showed a difference. In contrast, in females, Bamenda, localities of low altitude had a low parasite density, followed by Santa and Bambili (locality of high altitude). In female, the results quietly indicate that the altitude may affect the density of parasite in the population. Therefore, the absence of relationship between the malaria parasite density and the altitude of the living localities may due the habit of the males as due to the crisis in the region, males are forced to change regularly the house within the three localities.

Malaria was significantly more prevalent among patients that were 6–10 years in the entire study. Reports of similar studies conducted in Tanzania [24] and elsewhere in Cameroon [23] indicated patients of similar age groups with higher prevalence of malaria, respectively. Though the development of immunity to malaria is highly associated with frequency of exposure to the malaria parasite that is independent of altitude, there may be a switch in the age group of highest risk of malaria infection. This may also be due to over focused attention on malaria management among children 0–5 years. Across the three study areas (Bamenda, Santa and Bambili), there was no significant difference in the prevalence of malaria following the age categories. However, the parasite density in population was significantly different among children 0–5 years and adult of 36–60 years in the different localities. The general live condition of participants like type of farming, humidity etc. which vary following the altitude could possibly be the cause of this difference of malaria parasite density among people. Population living in Bambili of high altitude had high malaria parasite density. Majority of the patients in Santa, Bamenda, and in the entire study population suffered mild malaria infection with the exception of patients in Bambili where they were moderately infected. This suggests a possible impact of altitude on malaria severity and the results in the lowland areas of Bamenda were in conformity with a similar study in Tanzania that showed mild malaria prevalence among children in lowland area [23]. In addition, majority of the patients that tested positive for *P. falciparum* in the entire study were asymptomatic. An experimental study associated the expression of asymptomatic falciparum malaria and related severities with a switch in the host pro-inflammatory immune response against malaria parasite toward anti-inflammatory response [24]. This may lead to host tolerance to *P. falciparum* and possible suppression of the clinical presentation of malaria defined by temperature ≥ 37.5^0^C. This could further explain the significantly higher malaria parasitaemia observed among patients in Bambili and Bamenda though there was no significant variation in the mean malaria parasite density between these localities including the highland area of Santa. Malaria parasitaemia was significantly higher in females than in males in the entire study population. Considering the geographic settings of the study areas, male patients in Bamenda and Bambili recorded significant higher malaria parasitaemia than males in Santa. This similar trend was observed among females where those in Bambili and Santa had higher malaria parasitaemia than females in Bamenda. This suggests a possible gender based impact of altitude and climatic conditions on falciparum malaria parasitaemia.

It was observed that altitudinal variations significantly influenced the distribution of haemoglobin concentration, white blood cells and platelet counts among the populations living in the three different localities. The results revealed that people living in the Santa and Bambili highland areas had higher haemoglobin concentrations than in the Bamenda lowland area. These results were in conformity with reports of a study in the Middle East [25] that reported higher haemoglobin concentrations among people living at higher attitudes than in people in lowland areas. Generally, patients that tested positive for *P. falciparum* in the entire study registered lower haemoglobin concentration than patients that were negative and falciparum malaria patients in Bamenda were presented with mild anaemia as a result of lower haemoglobin concentration than patients in Santa and Bambili. Similar observations were recorded among malaria patients in Myanmar [20], the South West Region of Cameroon [22] and in Bamenda [26]. Females and patients 0–5 years significantly recorded high prevalence of anaemia than males and other age groups in all the study areas. This could be related to the biological development of the malaria parasite in parasitized red blood cells that correlated with the high burden of the disease among patients and the monthly physiological loss of blood in females leading to lower haemoglobin concentration in the infected populations. This leads to the suggestion that *P. falciparum* could be a contributing factor to the burden of anaemia especially in Bamenda and elsewhere.

Participants in Santa recorded higher WBC counts than those in Bamenda and Bambili. There was a similar trend among patients that tested positive for *P. falciparum* and infected patients also showed elevated levels of WBCs compared to non-infected participants. Though there was a significant increase in leucocyte levels among malaria patients in Santa, the mean WBCs level in all the populations of the different study areas were within normal and below the upper limit of normal range irrespective of their malaria status hence leukocytosis was absent in either of the populations. This suggests that altitudinal variation affects the level of WBCs among malaria patients but may not lead to serious pathologies associated with malaria infection. These suggestions coincide with results of [25] in the Middle East but conflicting with those of [5] in Cameroon where falciparum malaria patients were subjected to leucopoenia. This could be related to variations in the measurement scale and cut off points to define pathologies related to abnormal WBC counts in the study designs. In addition, platelet cell counts were significantly higher among malaria patients in highland areas than in lower land areas. Generally, malaria patients in all the study areas had significantly elevated platelet counts compared to non-infected participants. This was contrary to the reports of some studies [5] that associated malaria with platelet reduction (Thrombocytopenia).

Altitude had a significant impact on the serum myeloperoxidase (MPO) levels among patients infected with *P. falciparum* across the different study areas. Malaria patients in Bamenda and Bambili recorded higher MPO levels than patients in Santa. With regards to the study areas, patients that tested positive for *P. falciparum* had elevated levels of MPO than non-infected patients. The implication of MPO in falciparum malaria severity is not well elucidated but [13] reported that myeloperoxidase production increased during malaria parasite infection and correlated it with parasite clearance. This could possibly explain why majority of the infected patients in this study experienced mild and moderate malaria infection and variation in MPO levels could possibly be used as biomarker for investigating malaria infection in Mezam.

ITNs non-users, increase levels of WBCs and platelet counts were significant predictors of falciparum malaria. Patience presented with pyrexia, anaemia and increased levels of serum MPO levels had increased likelihood of malaria infection. This suggests that these parameters could be used as significant markers of *P. falciparum *.

## Conclusion

Prevalence of malaria infection was high and its distribution varied across altitudinal gradients in Mezam Division, Cameroon. The altitudinal variations had a significant impact on malaria parasitaemia. Clinical symptoms including haemoglobin concentration, WBC counts, platelet cell count and level of plasma MPO in malaria infected patients were significantly related to the altitudes. Therefore, significant attention should be given to malaria infection for both its proper diagnosis and treatment to prevent infection in Mezam Division. The role of environmental factors other than altitudes that facilitate the existence and transmission of malaria infection should also be further investigated.

## Supplementary Information

**Additional file 1.** Ethical clearance obtained for this study.

**Additional file 2.** Questionnaire form used for collection of demographic data.

**Additional file 3.** The raw data obtained from the field and the laboratory.

## Data Availability

All datasets on which the conclusions of the manuscript rely are presented in the paper.
