# Supplementary material for: Prevalence of malaria and associated clinical manifestations and myeloperoxidase amongst populations living in different altitudes of Mezam division, North West Region, Cameroon
Source: Malar J. 2023 Jan 19;22:20. doi: 10.1186/s12936-022-04438-6 (PMC9850770; doi:10.1186/s12936-022-04438-6)
Supplement: Supplementary file 1 — Additional file 1. Ethical clearance obtained for this study. [file 12936_2022_4438_MOESM1_ESM.pdf]

THE UNIVERSITY OF BAMENDA
P.O BOX 39 BAMBILI
North West Region
Tel.: (237) 233 360 033 / 233 366 029
Fax: (237) 233 366 030
Website: www.unibda.net

REPUBLIC OF CAMEROON
Peace – Work – Fatherland

MINISTRY OF HIGHER EDUCATION

ETHICAL REVIEW COMMITTEE/INSTITUTIONAL REVIEW BOARD
Chairperson: Prof. Vincent P. K. Titanji
Co-Chairperson: Prof. Theresia Nkuo-Akenji
Secretary: Prof. Helen Kuokuo Kimbi

Date 0 9 JUIL 2021

ETHICAL CLEARANCE ATTESTATION

Project Title: Altitudinal changes on malaria transmission: Variations in some blood parameters and myeloperoxidase levels among outpatients in mezam, Cameroon

Project identification Number: 2020/0224H/UBa/IRB

PI or Student's name(s): NGAHBORT BELTHINE FAKEH

The Ethical Review Committee of The University of Bamenda, after a study of your project proposal, and in keeping with existing regulations, hereby has no objections on ethical grounds provided the project is carried out as described in the protocol submitted to the Committee/Board. This attestation is non-transferable. Any changes to the protocol and /or personnel must get the approval of the Ethical Committee/Institutional Review Board otherwise the Ethical Clearance will be nullified.

This attestation which is valid for one year from the date of signature is issued to be presented as necessary.

Prof. Vincent P.K. Titanji
(Chairperson)

Prof. Helen K. Kimbi
(Secretary)
