# Supplementary material for: Prevalence of malaria and associated clinical manifestations and myeloperoxidase amongst populations living in different altitudes of Mezam division, North West Region, Cameroon
Source: Malar J. 2023 Jan 19;22:20. doi: 10.1186/s12936-022-04438-6 (PMC9850770; doi:10.1186/s12936-022-04438-6)
Supplement: Supplementary file 2 — Additional file 2. Questionnaire form used for collection of demographic data. [file 12936_2022_4438_MOESM2_ESM.pdf]

QUESTIONNAIRE

Topic; Please, honestly answer all the questions below

1. Sex
   ☐ Male ☐ Female
2. Age
   ☐ 3-10 ☐ 11-20 ☐ 21-30 ☐ 31-50 ☐ 50+
3. Marital status
   ☐ Single ☐ Married ☐ Widow/widower ☐ Divorce
4. Educational level
   ☐ No former education ☐ primary ☐ secondary level ☐ [illegible]gher education
5. Do you sleep under a treated mosquito net?
   ☐ Yes ☐ No
6. When last did you suffer from malaria
   ☐ Few weeks ago ☐ few months ago ☐ years ago
7. Are you currently on malaria treatment?
   ☐ Yes ☐ No
8. Do you know your HIV status?
   ☐ Yes ☐ No
9. Where do you live?
   ☐ Bamenda I ☐ Bamenda II ☐ Bamenda III ☐ Tubah
10. How long have you lived in this area?
    ☐ <3 years ☐ >3 years
11. How often do you travel out of North West?
    ☐ Monthly ☐ Every 6months ☐ Yearly
